# Targeted Knockdown of Overexpressed VEGFA or VEGF164 in Müller cells maintains retinal function by triggering different signaling mechanisms

**DOI:** 10.1038/s41598-018-20278-4

**Published:** 2018-01-31

**Authors:** Silke Becker, Haibo Wang, Aaron B. Simmons, Thipparat Suwanmanee, Gregory J. Stoddard, Tal Kafri, M. Elizabeth Hartnett

**Affiliations:** 1 John A. Moran Eye Center, University of Utah, Salt Lake City, UT USA; 2 Department of Internal Medicine, University of Utah, Salt Lake City, UT USA; 3 Gene Therapy Center, University of North Carolina at Chapel Hill, Chapel Hill, NC USA

## Abstract

Oxygen-induced retinopathy (OIR) upregulates Müller cell vascular endothelial growth factor A (VEGFA) that causes intravitreal neovascularization similar to severe retinopathy of prematurity (ROP). Safety concerns exist with anti-VEGF treatment for ROP. We evaluated long-term knockdown of Müller cell-VEGFA with short-hairpin RNAs to VEGFA or VEGF_164_ via subretinal lentivirus delivery (L-VEGFAshRNA, L-VEGF164shRNA) on retinal structure and function in a rat OIR model. Lectin-stained retinal flat mounts analyzed for areas of avascular/total retina (AVA) and intravitreal neovascular/total retina (IVNV) showed initial significantly reduced IVNV by L-VEGFAshRNA and L-VEGF164shRNA compared to control, luciferase-shRNA lentivirus, without late recurrence. Spectral-domain optical coherence tomography (OCT) and immunohistochemical sections (IHC) demonstrated changes in retinal layer thicknesses in L-VEGFAshRNA or L-VEGF164shRNA  compared to control. Ganzfeld electroretinograms were increased in L-VEGFAshRNA or L-VEGF164shRNA compared to control. Erythropoietin (EPO), brain-derived neurotrophic factor, glial-derived neurotrophic factor, nerve growth factor, neurotrophin-3 (NT-3) mRNAs were increased in L-VEGFAshRNA, but not L-VEGF164shRNA retinas. In cultured rat Müller cells, knockdown of VEGF upregulated NT-3 and EPO, whereas treatment with EPO activated neuroprotective signaling. Methods to reduce IVNV by selective knockdown of VEGFA, and particularly VEGF_164_, in Müller cells may have fewer deleterious effects than nonselective VEGFA inhibition to all cells in the retina.

## Introduction

Retinopathy of prematurity (ROP) is a leading cause of childhood vision loss and blindness worldwide^1^ and is increasing with survival of extremely preterm infants^2^. A major reason for vision loss is untreated severe ROP that leads to total retinal detachment. With premature birth, there is incomplete vascularization of the human infant retina with subsequent delayed physiologic retinal vascular development. When the infant is moved from supplemental oxygen to room air, poor oxygenation of the avascular retina stimulates disordered growth of retinal blood vessels into the vitreous rather than into the avascular retina. This intravitreal neovascularization can develop into severe, vision-threatening ROP^3^. Treatment of severe ROP is evolving with the use of anti-angiogenic agents that inhibit the bioactivity of vascular endothelial growth factor (VEGF) instead of standard care laser photocoagulation of the peripheral avascular retina^4^. VEGF is an important angiogenic factor involved in the pathogenesis of severe ROP^5^, but it is also important in the development of the retina^6^. Intravitreal neutralizing antibodies to VEGF also reduce VEGF in the bloodstream^7^ raising concerns of adverse effects on other developing organs in the preterm infant^8^. Besides these concerns on developing retina and organs, intravitreal anti-VEGF agents have altered the natural course of ROP with reports of reactivation of severe ROP and retinal detachment over a year after intravitreal injection^9^. Although clinical trials are testing lower doses of neutralizing VEGF antibodies in severe ROP^10^, a safe and effective dose remains elusive.

Studies in animal models representative of severe ROP in humans show that inhibition of VEGF with neutralizing intravitreal antibodies at certain doses effective at inhibiting retinopathy also reduce pup growth, reduce retinal capillary density and result in recurrent intravitreal neovascularization in association with activation of angiogenic signaling pathways in the retina^11,12^. We previously tested the hypothesis that targeted VEGF inhibition in Müller cells that overexpress VEGF would reduce aberrant angiogenesis into the vitreous. We developed short hairpin RNAs (shRNAs) to VEGFA under the control of a cell-specific promoter to inhibit overexpression of VEGFA in Müller cells^13^ and found reduced intravitreal neovascularization without adverse effects on physiologic retinal vascular development. However, knockdown of VEGFA led to cell death and thinning of the outer and inner nuclear layers in transduced retinas^14^.

An alternative splice variant of VEGFA, VEGF_164_, is overexpressed by repeated oxygen fluctuations, a stress associated with increased risk of ROP^15,16^. Mice lacking VEGF_164_ but engineered to express the other two rodent isoforms, VEGF_120_ and VEGF_188_, appeared to have normal retinal development^16^. These observations raise the possibility that inhibition of VEGF_164_ in Müller cells would be safer than inhibition of all VEGFA isoforms. Indeed, selective knockdown of VEGF_164_ in Müller cells with a lentivirus carrying VEGF_164_ shRNA driven by a cell specific promoter resulted in reduced intravitreal neovascularization in a rat oxygen-induced retinopathy (OIR) model^14^ without thinning of the outer nuclear layer in the short-term. In this study, we determined whether long-term inhibition of overexpressed VEGF_164_ specifically in Müller cells would be sufficient to inhibit intravitreal neovascularization without causing functional or structural loss to the retina and to explore effects of targeted VEGF knockdown in Müller cells on the retina.

## Results

### Course of Lentiviral Knockdown of VEGFA or VEGF_164_ on Intravitreal Neovascularization and Peripheral Avascular Retina in the Rat OIR Model

**Figure 1:**
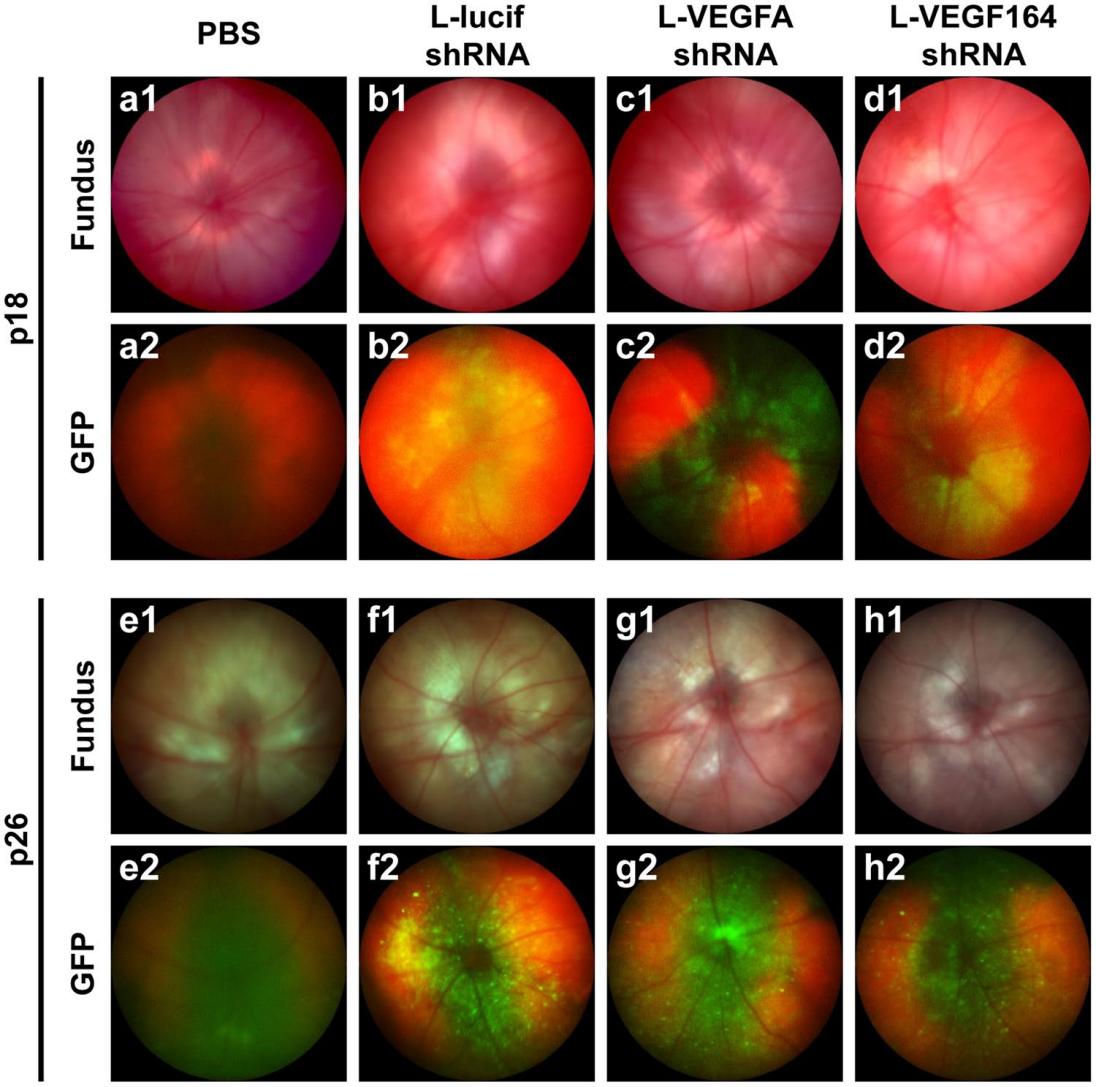
Representative fundus images and GFP expression at postnatal days (p)18 and p26 (**a**,**e**): after subretinal injection of PBS or after subretinal injection with pFmCD44 GFP lentiviruses with shRNA targeted against (**b**,**f**) luciferase (L-lucifshRNA), (**c**,**g**) VEGF-A (L-VEGFAshRNA), (**d**,**h**) VEGF_164_ (L-VEGF164shRNA).

**Figure 2:**
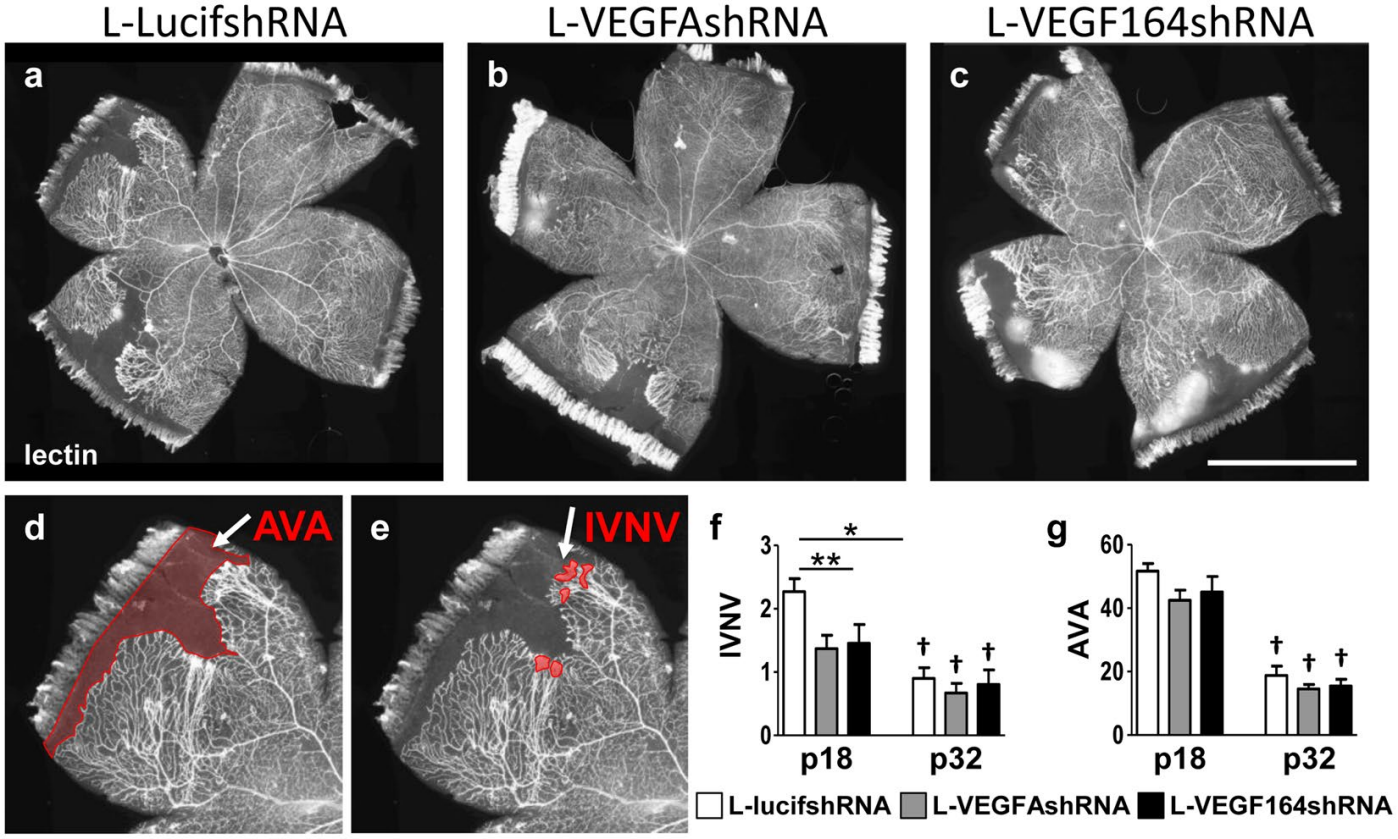
(**a–c)** Representative retinal flat mount images of lentivirus-treated retinas at p32 (Scale bar is 2 mm), (**d**–**e**). delineation of area with AVA and IVNV pointed by white arrows (**f**). IVNV, but not (**g**). AVA was significantly reduced at p18 after knocking down VEGF with either L-VEGFAshRNA or L-VEGF164shRNA (*p < 0.05, **p < 0.01 *vs*. L-lucishRNA; n = 5–8) (^†^p < 0.001 p32 *vs*. p18 for each treatment). There was no difference in AVA or IVNV between any of the groups at p32 (n = 12 for L-lucifshRNA, n = 9 for L-VEGFAshRNA and n = 10 for LVEGF164shRNA).

We first determined if knockdown of VEGFA or VEGF_164_ within Müller cells would adversely increase avascular/total retinal area (AVA) or lead to a recurrent intravitreal neovascular/total retinal area (IVNV) long-term in the OIR model since recurrence was seen following successful inhibition of IVNV with intravitreal neutralizing antibodies against rat VEGF164^11^. To determine this, lentiviruses were used to deliver vectors containing the cell specific CD44 promoter to drive the expression of shRNAs targeting VEGFA (L-VEGFAshRNA), VEGF_164_ (L-VEGF164shRNA) or luciferase (L-lucifshRNA) as a non-mammalian control within Müller cells. All the lentiviral constructs contained a GFP reporter to confirm successful transduction^14^. Efficiency was previously confirmed in HEK 293 GFP reporter cell lines expressing either rat VEGF_120_ to assess VEGFA knockdown or VEGF_164_ to assess the VEGF164 splice variant. Each reporter cell line was transfected with a plasmid DNA expressing VEGFA shRNA or VEGF164 shRNA and found to have reduced VEGF_120_ mRNA from VEGFA shRNA or VEGF_164_ mRNA by both plasmids compared to control luciferase shRNA plasmid^13^. Specificity of the lentiviruses had been confirmed *in vitro* by visualization of GFP in transduced rat Müller cells (rMC-1 cells, kindly provided by V. Sarthy) with no GFP visualized in human retinal microvascular endothelial cells (hRMVECs) and *in vivo* by GFP visualization consistent with transduced Müller cell endfeet with the Micron IV (Fig. 1) and confirmed on glutamine synthetase and GFP-colabeled cryosections^13,14^. Only eyes with confirmed Müller cell transduction were included in subsequent analyses. At p18, a time point of IVNV in the rat OIR model, significant inhibition of IVNV was detected (**P = 0.002 L-VEGFAshRNA and *P = 0.014 L-VEGF164shRNA vs. L-lucifshRNA) with no adverse effects to AVA (Fig. 2), as previously found^14^. At p32, a time point when the rat OIR model naturally undergoes regression of IVNV and ongoing vascularization of the peripheral avascular retina, there was no difference in AVA or IVNV between L-lucifshRNA control and either L-VEGFAshRNA or L-VEGF164shRNA (Fig. 2), providing evidence that Müller cell specific knockdown of VEGFA or VEGF_164_ did not lead to recurrent retinopathy.

### Müller cell knockdown of VEGFA or VEGF164 thins outer nuclear and photoreceptor layers

**Figure 3:**
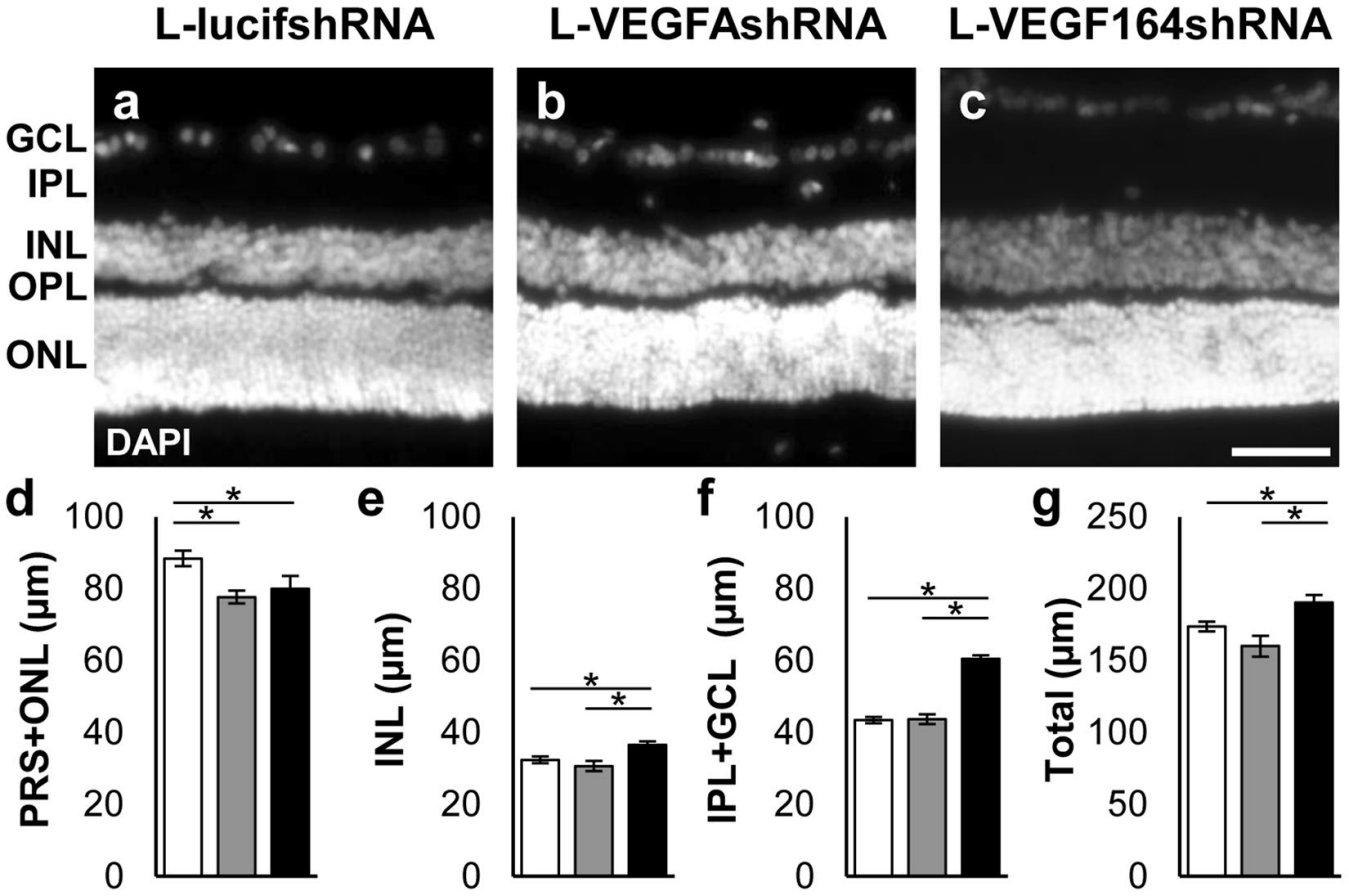
(**a**–**c**) Representative micrographs of cryosections stained with DAPI nuclear stain. (**d**) Thickness of the photoreceptor and outer nuclear layer (PRS + ONL) was significantly reduced in retinas transduced with L-VEGFAshRNA or L-VEGF164shRNA. **e–g**) Thicknesses of the inner nuclear layer (INL), inner plexiform layer and ganglion cell layer (IPL + GCL) and total retina were significantly increased in retinas transduced with L-VEGF164shRNA (*p < 0.05 *vs*. L-lucishRNA and *vs*. L-VEGFAshRNA; n ≥ 16 images). Scale bar in d = 25 µm.

**Figure 4:**
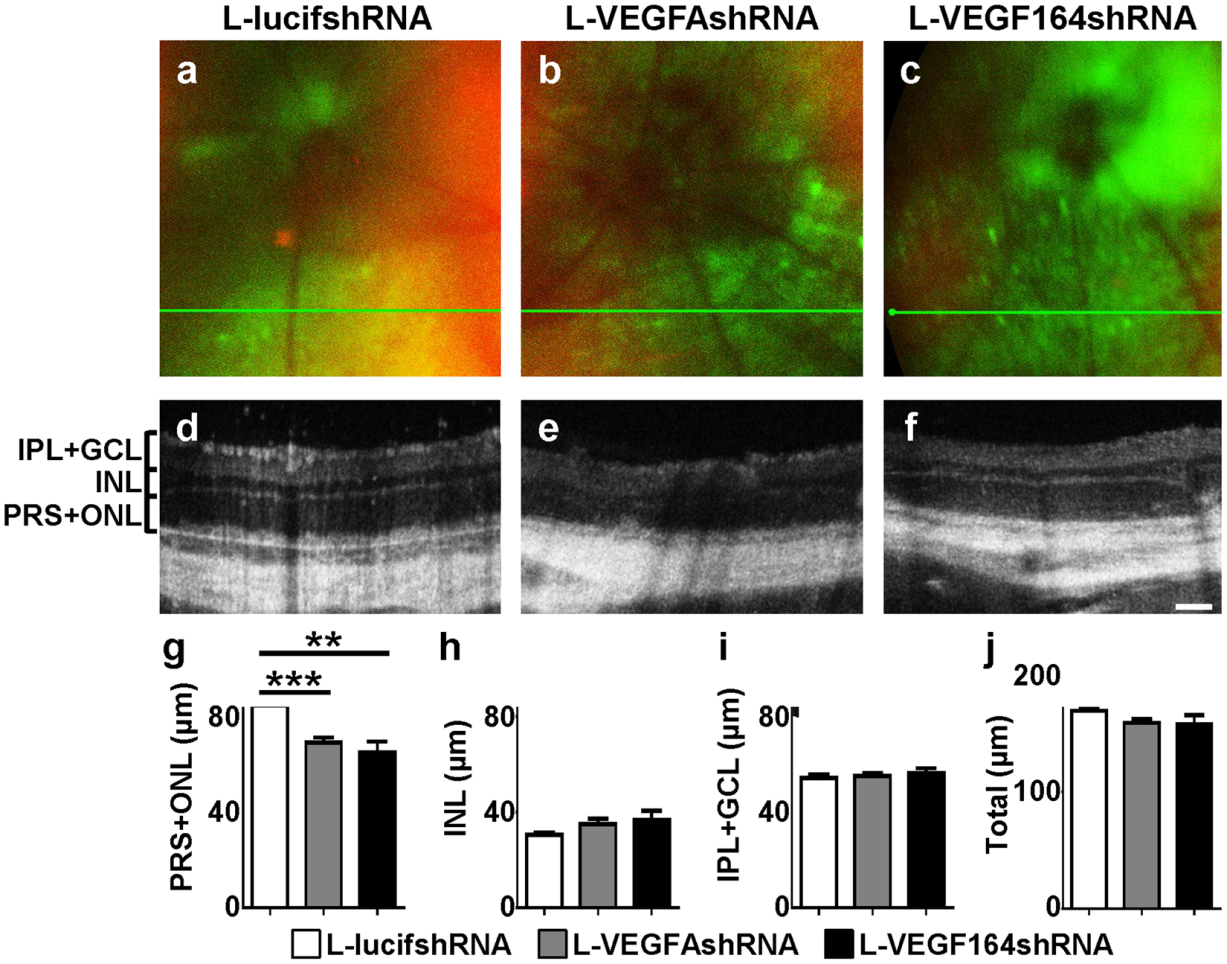
(**a–c)** MicronIV images showing GFP-transduced areas sampled by OCT. (**d–f)** Representative OCT images. (**g)** Thickness of the PRS + ONL measured by OCT was significantly reduced in GFP-positive areas of retinas transduced with L-VEGFAshRNA (***p < 0.001, n = 4) or L-VEGF164shRNA (**p < 0.01, n = 6) compared to L-lucifshRNA (**p < 0.01, ***p < 0.001 *vs*. L-lucishRNA; n = 9). (**h–j**). No difference was observed in INL, IPL + GCL, or total retina thickness between any of the groups. Scale bar in f = 100 µm.

Previously, TUNEL positive cells were increased in rat OIR transduced retinas at p18 with later thinning of retinal layers measured on immunohistochemical (IHC) sections at p25 in L-VEGFAshRNA but not L-VEGF164shRNA treated eyes^14^. Therefore, we predicted retinal structure would be altered at p32 in L-VEGFAshRNA-, but not in L-VEGF164shRNA- treated eyes. However, we detected thinning of photoreceptor and outer nuclear layers (PRS + ONL) by IHC in both L-VEGFAshRNA and L-VEGF164shRNA treated eyes compared to L-lucifshRNA controls measured at p32 (Fig. 3a–d, p = 0.001 L-VEGFAshRNA; p = 0.049 L-VEGF164shRNA vs. L-lucifshRNA). In contrast, the inner nuclear (INL) was thicker in L-VEGF164shRNA compared to either L-VEGFAshRNA or L-lucifshRNA (Fig. 3e, p = 0.001 L-VEGFAshRNA; p = 0.004 L-lucifshRNA vs. L-VEGF164shRNA), as were the inner plexiform and ganglion cell layers (IPL + GCL) (Fig. 3f, p = 0.011 L-VEGFAshRNA; p = 0.003 L-lucifshRNA vs. L-VEGF164shRNA) and total retina (Fig. 3g, p = 0.002 L-VEGFAshRNA; p = 0.016 L-lucifshRNA vs. L-VEGF164shRNA). There was little evidence of cell death determined by TUNEL + staining in any group including subretinal PBS control (data not shown), suggesting that the lentivirus itself was not toxic at this time point. Additionally, retinal structure was analyzed *in vivo* with spectral-domain optical coherence tomography (OCT). For this analysis, only GFP-positive retinas transduced with L-VEGFAshRNA, L-VEGF164shRNA or L-lucifshRNA were imaged and layer thicknesses measured (Fig. 4a–f). There was significant thinning of the photoreceptor and outer nuclear layers (PRS + ONL) in L-VEGFAshRNA and L-VEGF164shRNA treated eyes compared to L-lucifshRNA control (Fig. 4g, P < 0.001 L-VEGFAshRNA and P = 0.003 L-VEGF164shRNA vs L-lucifshRNA), but the thicknesses of the INL (Fig. 4h), IPL + GCL (Fig. 4i) and total retina (Fig. 4j) were not significantly different in L-VEGFAshRNA or L-VEGF164sRNA compared to L-lucifshRNA. Altogether, the findings suggest that knockdown of VEGFA or VEGF_164_ in Müller cells leads to thinning of the photoreceptor and outer nuclear layers, and that knockdown of VEGF_164_ in Müller cells may increase the thicknesses of the GCL, IPL and INL based on IHC. IHC recorded retinal thickness in both nontransduced and transduced retinal regions, whereas OCT measurements only included transduced retina, which may account for differences in the thickness measurements of the INL and IPL + GCL by each method. To assess if the lentivirus itself caused thinning, we compared OCTs of L-lucifshRNA to subretinal PBS treated eyes and found no difference in any layer thicknesses (data not shown), suggesting  that the lentivirus alone was not principally causing the structural differences observed.

### Müller cell knockdown of VEGF or VEGF_164_ improves retinal function

**Figure 5:**
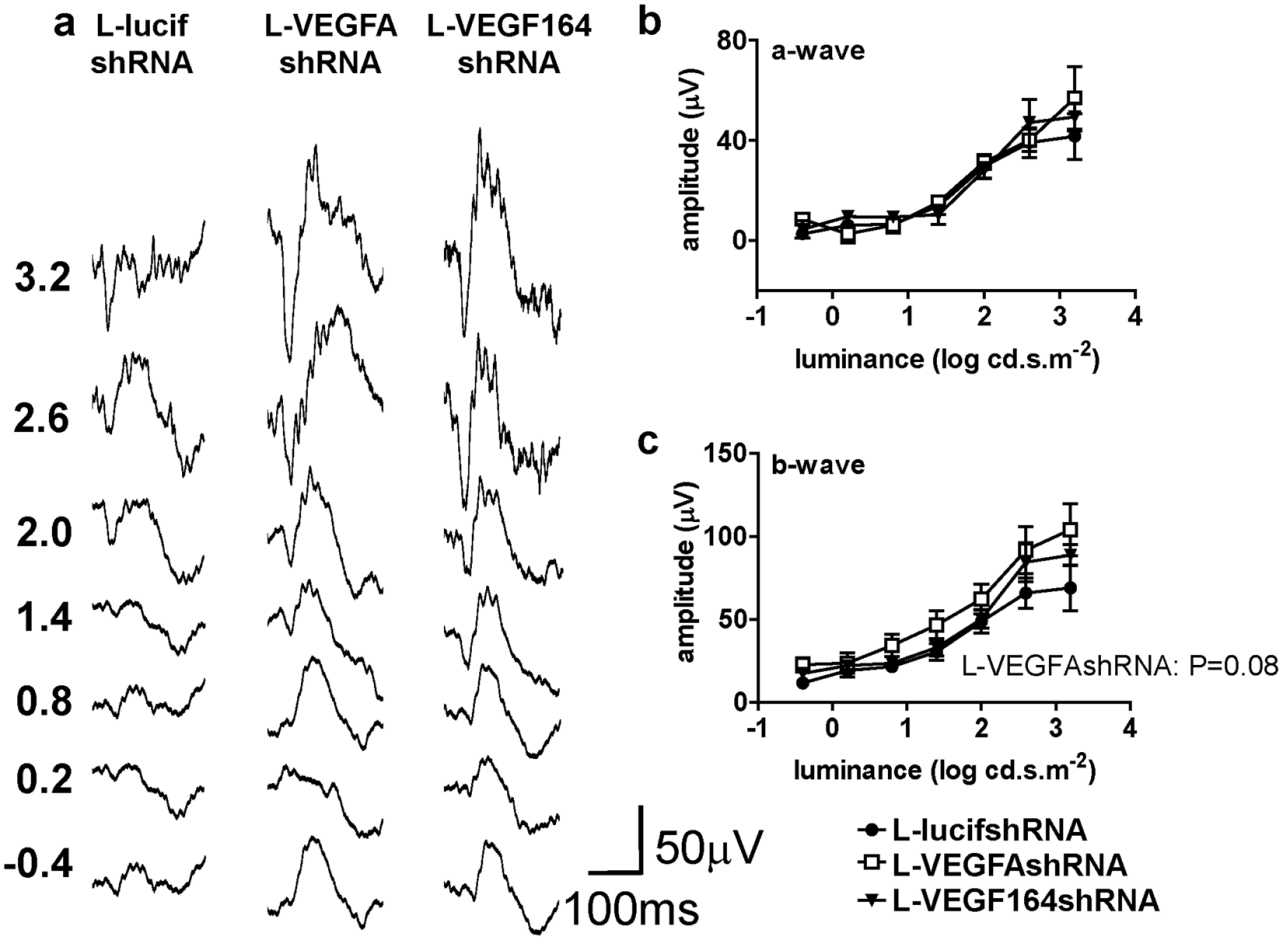
(**a**) Example traces of scotopic focal ERGs in L-lucifshRNA, L-VEGFAshRNA or L-VEGF164shRNA treated animals at p32 (**b**,**c)**. The amplitudes of the a-wave and b-wave of the scotopic focal ERGs were not significantly changed by L-VEGFAshRNA (n = 10) or L-VEGEF164shRNA VEGF_164_ (n = 13) compared to L-lucifshRNA (n = 7).

**Figure 6:**
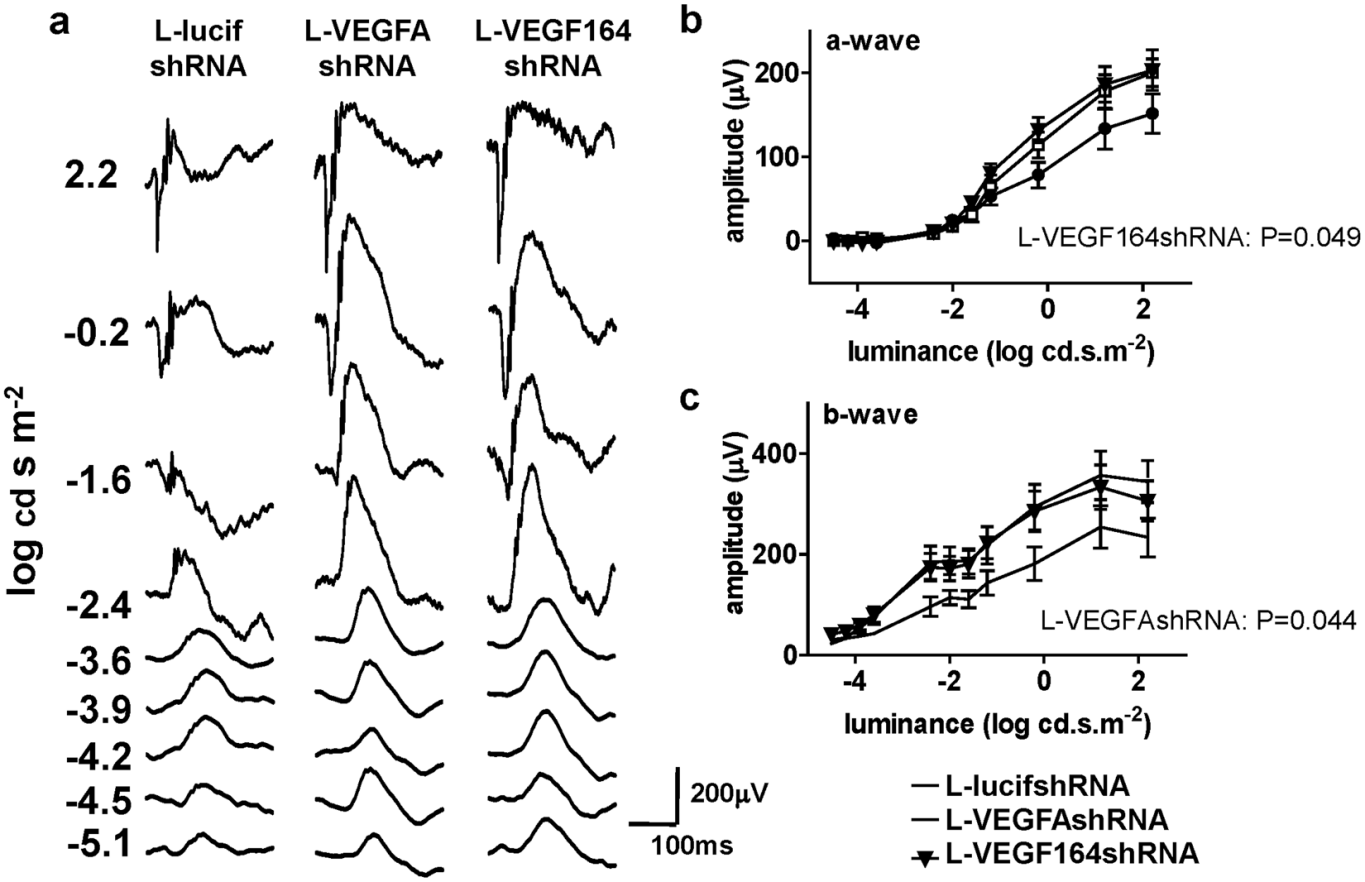
(**a**) Example traces of scotopic Ganzfeld ERGs in L-lucifshRNA, L-VEGFAshRNA or L-VEGF164shRNA treated animals at p32. (**b**) The amplitude of the scotopic a-wave in the Ganzfeld ERG was increased in retinas transduced with L-VEGF164shRNA compared to L-lucifshRNA (p = 0.049 L-VEGF164shRNA *vs*. L-lucishRNA; n = 12 and n = 10, respectively). (**c**) The amplitude of the b-wave in the scotopic Ganzfeld ERG was significantly increased in retinas transduced with L-VEGFAshRNA compared to L-lucifshRNA (p = 0.044 L-VEGFAshRNA *vs*. L-lucishRNA; n = 11 and n = 10, respectively).

Inhibition of VEGF has been associated with death of retinal cells, such as photoreceptors^14^, Müller cells^17^ and retinal ganglion cells^18^. Therefore, thinning of the photoreceptor cell layers by L-VEGFAshRNA or L-VEGF164shRNA treatments would be predicted to be associated with reduced retinal function measured as reduced a-wave amplitudes by electroretinography. Focal electroretinograms (ERGs) were performed in transduced, GFP-positive retina. Compared to L-lucifshRNA treated eyes, there was no significant difference in focal ERG a- or b-wave amplitudes in L-VEGFAshRNA (P = 0.731 and P = 0.08, respectively) or L-VEGF164shRNA (P = 0.322 and P = 0.309, respectively) (Fig. 5a–c). This suggested that Müller cell transduction of L-VEGFAshRNA or L-VEGF164shRNA did not adversely affect retinal function. In contrast to the focal ERG, which was used to measure function in the transduced retina only, the full-field ERG measures function over the entire retina that includes transduced and non-transduced regions. Compared to L-lucifshRNA, the full-field ERG a-wave and b-wave amplitudes were larger in the L-VEGFAshRNA or L-VEGF164shRNA compared to L-lucifshRNA treated eyes; the a-wave for photoreceptor function was significantly larger in eyes transduced with L-VEGF164shRNA(p = 0.049) (Fig. 6a,b); and the b-wave for inner retinal and bipolar cell function was significantly larger in eyes transduced with L-VEGFAshRNA (P = 0.044) (Fig. 6a and c). These findings suggested that specific knockdown of Müller cell VEGFA or VEGF_164_ led to improved overall retinal function at p32 compared to control L-lucifshRNA.

### Müller cell specific VEGF knockdown increases neurotrophic factors and activates survival signaling

**Figure 7:**
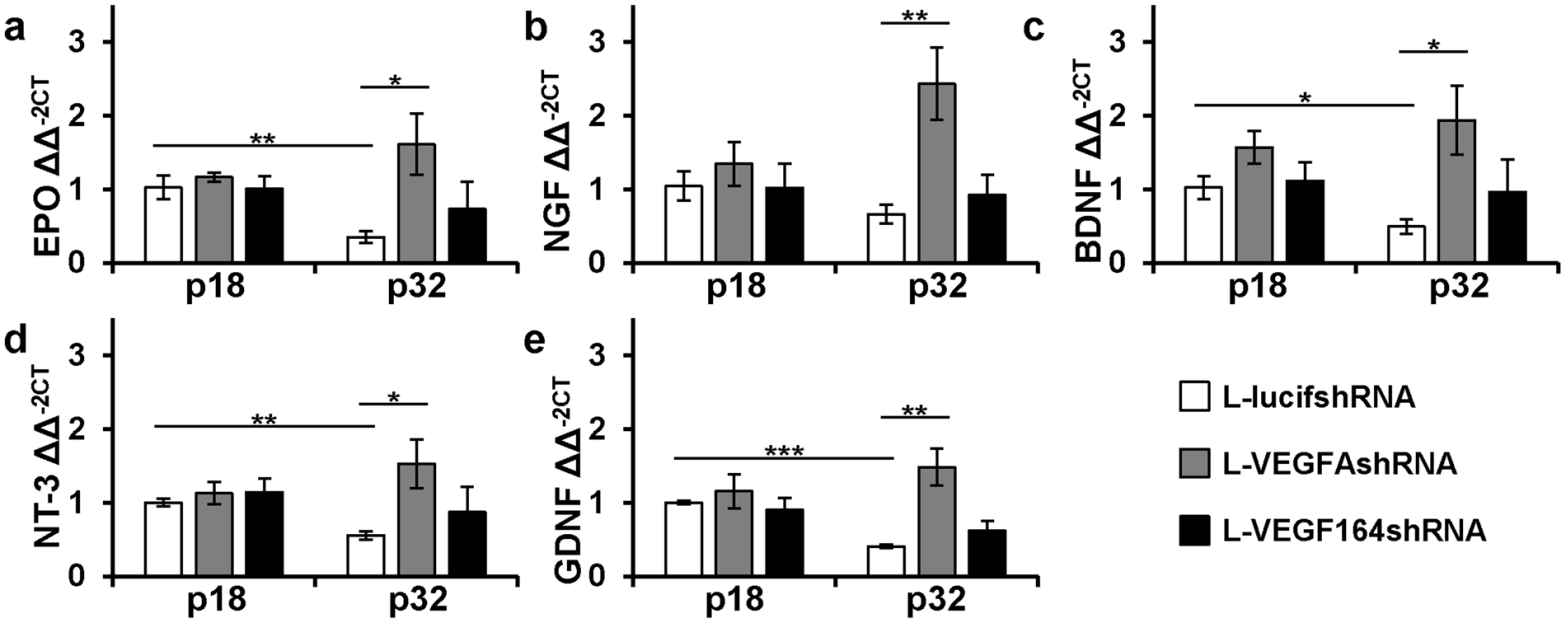
A significant increase in the mRNA expression of the neurotrophic factors: (**a**) EPO, (**b**) NGF, (**c**) BDNF, (**d**) NT-3 and (**e**). GDNF was observed in retinas after injection of L-VEGFAshRNA compared to L-lucifshRNA at p32 (*p < 0.05, **p < 0.01 and ***p < 0.001 *vs*. L-lucishRNA at p18; n = 4–5). EPO, BDNF, NT-3 and GDNF were decreased in L-lucifshRNA at p32 compared to p18.

**Figure 8:**
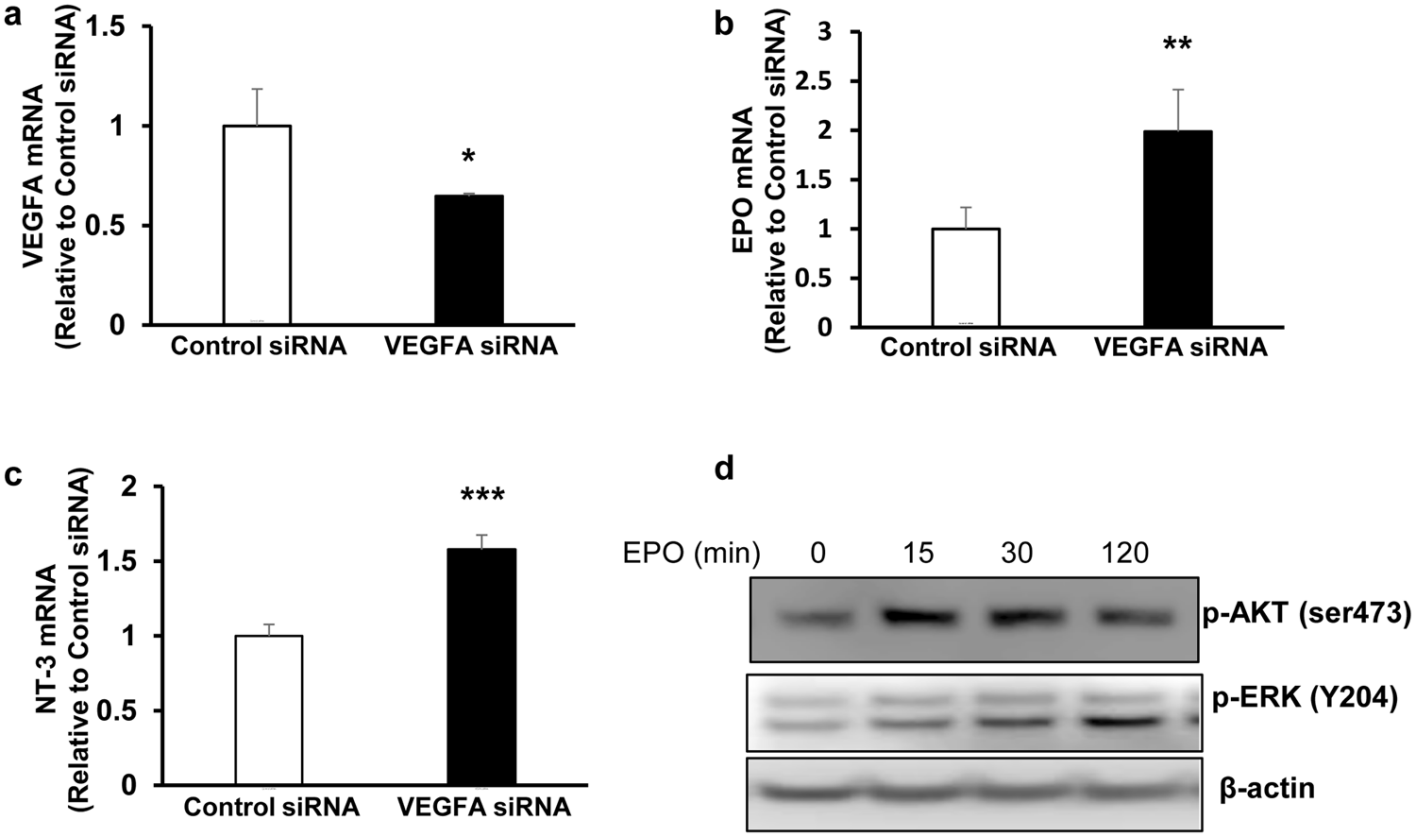
Knockdown of VEGFA in rat Müller cells *in-vitro* increases expression of neurotrophic factors and activates survival signaling. a-c: Real time PCR of (**a**) VEGFA, (**b**) EPO and (**c**) NT-3 mRNA in rat Müller cells transfected with siRNA targeting rat VEGFA or negative control siRNA (*p < 0.05, **p < 0.01 and ***p < 0.001 vs. Control siRNA; n = 3); Western blots of: (**d**) phosphorylated AKT (p-AKT) and (p-ERK) in rat Müller cells treated with recombinant EPO (1 unit/ml) for 15, 30 and 120 mins (n = 3) (p-AKT and p-ERK were probed on different gels. For each gel, either p-AKT or p-ERK was probed and then reprobed for β-actin so that β-actin was probed for each gel. p-AKT and its β-actin were cropped from the same gel. P-ERK and β-actin were cropped from the same gel. The full length of the gels were included in Supplemental Fig. 3).

Inhibition of VEGF with intravitreal neutralizing antibodies can increase Müller cell expression of erythropoietin (EPO) through a mechanism involving STAT3 activation and nuclear translocation^19^. EPO has angiogenic and neuroprotective functions^20^. Since VEGF is neuroprotective and secreted, it is possible that reduced VEGF expressed by Müller cells may trigger other mechanisms in Müller and other retinal cells throughout the retina to express neuroprotective factors in order to restore retinal function. To address the hypothesis that specific Müller cell knockdown of VEGFA or VEGF_164_ leads to increased expression of protective factors, we measured mRNAs at p18 and p32 in retinas for several neurotrophic and neuroprotective factors produced by Müller cells. In comparison to L-lucifshRNA-treated eyes, EPO (*P = 0.0248), nerve growth factor (NGF, **P = 0.0098), brain-derived neurotrophic factor (BDNF, *P = 0.0234), neurotrophin-3 (NT-3, *P = 0.0271) and glial cell-derived neurotrophic factor (GDNF, **P = 0.0053) were increased in L-VEGFAshRNA-treated but not in L-VEGF164shRNA-treated retinas at p32 (Fig. 7). EPO, BDNF, NT-3 and GDNF expression was reduced in L-lucifshRNA treated eyes at p32 compared to p18. We then addressed the question if Müller cells, knocked down for VEGF, expressed neurotrophic factors in an autocrine manner. Isolated and cultured primary rat Müller cells (passages 3–5) were transfected with VEGF siRNA or control siRNA and found to have 40% knockdown (Fig. 8a, p = 0.013). Real-time PCR performed 48 hours after transduction demonstrated EPO (Fig. 8b, p = 0.008) and NT-3 (Fig. 8c, p = 0.0002) significantly increased. The other neurotrophic factors found increased in retinal lysates from the rat OIR model were not significantly affected in cultured rat Müller cells stimulated with EPO.

We then asked if EPO would activate signaling pathways in Müller cells that were important for retinal cell survival. Rat Müller cells were stimulated with EPO, and activation of extracellular signal regulated kinase (ERK) or protein kinase B (PKB/AKT) was determined as phosphorylated ERK (p-ERK) or p-AKT on western blots. Both p-ERK and p-AKT (Fig. 8d) were induced 15 minutes after EPO treatment and maintained through 120 minutes.

## Discussion

Excessive VEGF signaling disorders developmental angiogenesis in the preterm infant retina and contributes to severe, vision-threatening ROP^3^. However, VEGF is also important in retinal^6,17,21^ and other organ development^8^. The infants at greatest risk of severe ROP in the US are often extremely premature infants, born younger than 28 weeks gestational age and less than 1000 g birth weight. The Extremely Premature Infants in Sweden Study (EXPRESS) reported that infants under 28 weeks gestational age who never developed ROP still had reduced ERG function and vision at age 6.5 years^22,23^. These infants also may be the most vulnerable to the effects of VEGF inhibition, because as young, very premature infants, less neural development has occurred than in older premature infants who have already met some milestones. There are slight differences in ocular volumes between very premature and older premature infants, making a similar intravitreal dose of anti-VEGF agent more concentrated in the younger, smaller eye. Furthermore, it is not safe to measure the amount of VEGF in the infant vitreous in order to adjust the dose of anti-VEGF agent. Effects of a single anti-VEGF intravitreal injection in infants include persistent avascular retina and later recurrent severe ROP^24^. Therefore, greater understanding of the relationships among glia, the neural retina and vasculature become increasingly important as ever younger and smaller premature infants are surviving and developing severe ROP.

Anti-VEGF agents enter the circulation and are diluted in the blood volume, which is about 6% of the total body weight. Therefore, in different sized infants, the concentration of circulating anti-VEGF agent after a single intravitreal dose may vary. Levels of VEGF in the circulation can be reduced for 2 months following a single dose of anti-VEGF^7^. Efforts are needed to modify anti-VEGF dose or agent^10^, or to target anti-VEGF strategies to cells that overexpress it, or to target signaling pathways involved from overexpessed VEGF. We wished to learn more about the mechanisms and safety of knocking down overexpressed VEGFA or the splice variant, VEGF_164_, in Müller cells at later time points. The importance and role of Müller cell-derived VEGF in IVNV has been demonstrated in mouse and rat OIR models^13,25^. In this study, our goals were to assess longer-term effects of targeted VEGF knockdown on retinal structure and function in an experimental model that is representative of many of the features of human ROP. As previously reported, IVNV was inhibited at p18 by either experimental lentivirus compared to control without an increase in AVA^14^ and no increase in IVNV or AVA at p32. IVNV and AVA persisted in all groups at p32, but the magnitudes were not different from other studies in which subretinal or ocular injections were used^26^. However, we found thinning of the photoreceptor and ONL layers in L-VEGFAshRNA or L-VEGF164shRNA compared to L-lucifshRNA control suggesting that Müller cell knockdown of VEGFA or VEGF_164_ may have harmful effects on photoreceptors. In other models, VEGF has been found to be important for photoreceptor health and survival^17^. Thickening of the INL and IPL + GCL observed by IHC following L-VEGF164shRNA may be due to incomplete knockdown of all VEGF isoforms in the retina by this treatment. In particular, soluble VEGF_120_, which is not targeted by L-VEGF164shRNA, may diffuse to and affect the entire retina, not only to local areas in the case of cell-associated, longer splice variants. We did not observe a compensatory upregulation of VEGF_120_ and VEGF_164_ mRNA by either L-VEGFAshRNA or L-VEGF164shRNA treatments.

In contrast to predicted, function was not worse in transduced areas of L-VEGFAshRNA and L-VEGF164shRNA treated eyes measured by focal ERG. Furthermore, full-field ERG amplitudes were increased in L-VEGFAshRNA and L-VEGF164shRNA treated eyes compared to L-lucifshRNA. This raised the possibility that expression of neuroprotective factors triggered by specific knockdown of VEGFA or VEGF_164_ in Müller cells improved function of the neural retina. Higher a- and b-wave amplitudes observed in the Ganzfeld ERG indicate that factors, which likely mediate this improvement, are able to diffuse within the retina to non-transduced areas. Although L-VEGF164shRNA did not result in detectable upregulation of any of the neuroprotective factors or EPO investigated in this study, increased ERG function may be through different pathways or protective factors, including expression of VEGF splice variants that were not affected by VEGF_164_ knockdown.

Better retinal function was observed following treatment with L-VEGFAshRNA or L-VEGF164shRNA compared to L-lucifshRNA control in the full field scotopic ERG despite thinning of the photoreceptor layer. It is not yet clear the reason for this observation, but  may depend, in part, on the  degree of knockdown and timing of ERG analysis. A previous study in the mouse OIR model^27^ reported that retinal function did not always correlate well to ONL thickness. In the rat OIR model, despite similar thicknesses of the photoreceptor and inner nuclear layers, subretinal PBS injection resulted in increased b-wave amplitude compared to pilot hole without injection^26^. These findings suggest that the change in ONL thickness does not directly reflect visual function or that the timing of the ERG has not aligned with the structure of the retina.

Neurotrophic factors are known to rescue photoreceptors from degeneration^28^. A previous report showed that an intravitreal neutralizing antibody to VEGF increased Müller cell EPO expression in the rat OIR model^19^. EPO also has proposed neuroprotective effects^20^. Following L-VEGFAshRNA transduction, EPO, GDNF, NT-3, NGF and BDNF were all significantly upregulated at p32 but not at p18. These findings supported the notion that long-term knockdown of VEGFA activated neurotrophic factors in the retina, whereas knockdown of VEGF_164_ (and potentially VEGF_188_ splice variant) did not. Both L-VEGFAshRNA and L-VEGF164shRNA thinned the photoreceptor and ONL layers, but only L-VEGFAshRNA led to increased expression of neurotrophic factors. On the other hand, L-VEGF164shRNA thickened the INL, IPL and GCL without increased expression of neurotrophic factors. These findings may represent differences in the degree of VEGF knockdown or timing of events related to induced protective pathways on neural processing in the retina, as the INL and IPL involves many connections among retinal neurons and glia. For example, if early upregulation of neurotrophic factors occurred following L-VEGFAshRNA or L-VEGF164shRNA Müller cell transduction, some protective effects such as increased ERG amplitudes with thickening of retinal layers may have occurred at the later p32 time point. There also may be differential effects from knockdown of VEGF splice variants. Presence of the two splice variants VEGF_120_ or VEGF_188_ is sufficient for normal retinal vascular development^16^, but VEGF_164_ was associated with inflammatory findings in experimental retinal models^16^ and was upregulated by repeated fluctuations in oxygenation^15^, a risk of severe ROP. Therefore, inhibition of VEGF_164_ may reduce pathology without being as harmful to the retina as inhibition of VEGFA. VEGF_120_ is transcribed from the VEGFA gene by variant splicing, translated and is secreted, whereas VEGF_164_ has a heparin-binding domain rendering it cell-associated also. Great concentrations of secreted VEGF may have provided some protective effects manifested as thickened layers of the retina with less stimulus for neurotrophic factor expression in the L-VEGF164shRNA treated eyes. An aptamer to inhibit the human analog, VEGF_165_, did not appear to have as beneficial an effect in studies of neovascular AMD^29^, but other methods of inhibition may be more effective in OIR or ROP. More study is needed. IHC was performed in the central retina in all samples. However, some central retinal sections included in thickness measurements likely involved non-transduced retina. In contrast, OCTs were performed in transduced retina only as this was visualized as GFP-positive. Some of the discrepancy between IHC and OCT measurements may be due to such differences in measurements by IHC vs. OCT.

Knockdown of VEGF upregulated NT-3 and EPO in cultured Müller cells. Other cells besides Müller cells may have been stimulated to produce other neurotrophic factors when Müller cell-expressed VEGF was inhibited *in vivo*. Also, NT-3 can bind tyrosine kinase receptors (TrKC and TrKB) and trigger signaling pathways that indirectly affect neuroprotective factor expression in other cells at later time points. NT-3 may have been upregulated in Müller cells following VEGF knockdown as both factors were found to be neuroprotective for photoreceptors^28^. In addition, EPO also has been reported to trigger neuroprotective pathways^30^ and to increase BDNF in Müller cells^31^. In cultured Müller cells stimulated with EPO, no increased neurotrophic factor expression was detected, but EPO activated p-AKT and p-ERK, kinases that are involved in neuroprotective signaling^30^. Finally, there may be differences between lentiviral transduction of VEGFA shRNAs and transfected cultured Müller cells with VEGF siRNA.

In conclusion, our study suggests that inhibition of VEGFA splice variants in Müller cells can cause structural abnormalities in the retina but also functional improvement of the ERG via expression of neuroprotective factors from Müller and other retinal cells known to protect photoreceptors. By selectively targeting VEGF_164_ in Müller cells, protective mechanisms may be through a different mechanism including by expression of secreted shorter forms of VEGFA. Selective knockdown of VEGFA or VEGF_164_ in Müller cell may reduce potential adverse effects from nonselective VEGFA inhibition to all cells in the retina, as what occurs with neutralizing intravitreal antibodies to VEGF. Additional studies to test the roles of VEGFA, VEGF_164_ and EPO on vascular, neural and glial mechanisms in the retina and the effects on functioning and development are warranted. These studies provide insight into the effects of inhibiting VEGF in retinopathy on retinal structure and function.

## Materials and Methods

### Lentivector-Driven shRNA

As previously reported^14^, short hairpin RNAs targeting firefly luciferase (accession number M15077, non-mammalian control, luciferase shRNA), rat VEGF_164_ (accession number AF260425, VEGF_164_ shRNA) and rat VEGFA (accession number NM_031836, VEGF-A shRNA) were embedded into a microRNA (miR-30) context. The miR-30/shRNAs were cloned into a lentiviral transfer vector under transcriptional control of a CD44 promoter (pFmCD44.1 GW), with green fluorescent protein (GFP) as a reporter gene. Lentiviral vectors were generated and concentrated by transient three-plasmid transfection into 293 T cells and ultracentrifugation, respectively, as described earlier^32^.

### Cell culture and transfection

Primary rat Müller cells were isolated from one litter of 8 day old Sprague Dawley rat pups using a protocol adapted from Hicks and Courtois^33^. Briefly, eyes were enucleated and incubated in serum-free Dulbecco’s Modified Eagle’s medium (DMEM)/GlutaMAX at room temperature overnight followed by digestion at 37 °C in 0.001 g/mL Trypsin and 70 U/mL collagenase in serum-free DMEM for 20 min and wash in DMEM + 10% fetal bovine serum (FBS). Retinas were then dissected in serum-free DMEM and dispersed by pipetting until the retinal cell suspension was homogenous. Cells were re-suspended in 10% FBS in DMEM, seeded into a 10 cm tissue culture dish and incubated in 5% CO_2_ at 37 °C. After 6 to 7 days, cultured cells were washed with PBS and the cells that did not have Müller cell morphology were removed by rubbing with an 18 G cannula. Presence of Müller cell markers vimentin and glutamine synthetase by immunostaining were used to confirm cell identity (Supplemental Fig. 1). Cells were maintained in 10% FBS, DMEM/GlutaMAX and used from passages 3 to 5.

To knockdown VEGFA, rat primary Müller cells, were transfected with silencer selective siRNA targeting rat VEGFA, or a silencer selective negative control siRNA using lipofectamine 2000 (Invitrogen, Carlsbad, CA). Within 48 hours of transfection, cells were collected for RNA analysis.

### RNA isolation, reverse transcription and quantitative real-time PCR of neurotrophic factors

RNA from retinas or rat primary Müller cells were isolated using a TRI reagent (Sigma Aldrich, St. Louis, MO) and RNA concentrations were determined with a Nanodrop 2000 Spectrophotometer (Nanodrop, Wilmington, Delaware). RNA was reverse transcribed using High-Capacity cDNA Reverse Transcription kit (Applied Biosystems, Foster City, CA). Expression of mRNA coding for rat VEGFA, EPO, NT-3, BDNF, GDNF, NGF and the housekeeping gene GAPDH was measured using specific PCR primers (HSC Core, University of Utah, Salt Lake City, Utah) and SYBR Green Mastermix (Applied Biosystems) in a Bio-Rad CFX Connect Real-Time System (Bio-Rad, Hercules, California). Expression levels for all genes were normalized to the mean value of the internal control, GAPDH. Each sample was run in duplicates and changes in gene expression were displayed as fold difference of ΔΔ2^−CT^ normalized to samples treated with luciferase shRNA or control siRNA. Sequences of the primers used in quantitative real time PCR were listed below:

Rat VEGFA GCTCCTTCACTCCCTCAAATTA (Forward)

   GGTCTCTCTCTCTCTCTCTCTTC (Reverse)

Rat EPO:     AGCAGGAGAGACTGAGAGAA (Forward)

    CCTTGACTACATAGCGAGTTCC (Reverse)

Rat NT-3:   CATAAGAGTCACCGAGGAGAGTA (Forward)

  GATCTCTCCCAACACTGTAACC (Reverse)

Rat BDNF     GCACAGAAAGCTCCTGATAGT (Forward)

    CCAGCTTGACTTCTCCTAACC (Reverse)

Rat GDNF    CTACGAAACCAAGGAGGAACTG (Forward)

   CCTTGTCACTTGTTAGCCTTCT (Reverse)

Rat NGF   CTCTGAGGTGCATAGCGTAAT (Forward)

  CTGGGACATTGCTATCTGTGTA (Reverse)

Rat GAPDH CATCTCCCTCACAATTCCATCC (Forward)

GAGGGTGCAGCGAACTTTAT (Reverse)

### Animals

Animal care and procedures were in accordance with the ARVO Statement for the Use of Animals in Ophthalmology and Vision Research and approved by the Institutional Animal Care and Use Committee (IACUC) at the University of Utah. The authors confirm that the experimental protocols were approved by IACUC and the Institutional Biosafety Committee of the University of Utah. Timed-pregnant Sprague Dawley dams were purchased from Charles River (San Diego, CA). Pups were delivered naturally between embryonic days 21 and 23. Animals were maintained under a 12 hour dark/light cycle and had free access to food and water.

### Oxygen-induced Retinopathy

The rat 50/10 model of OIR is a well-characterized animal model of ROP^12,34^. Within 6 hours of birth litters were supplemented to contain 12–16 pups, placed into a controlled oxygen chamber (Oxycycler, Biospherix, Parish, New York) with a dam and exposed to oxygen cycling between 50% and 10% O_2_ every 24 hours for 14 days. Animals were placed into room air from p14 to p32. The model creates features of OIR pathology (IVNV and AVA) by postnatal day 18 to 20, with regression of abnormal features and physiologic vascularization of the avascular retina^35^. At p32, untreated animals had IVNV of 0.26 ± 0.14 SEM % and AVA of 5.62 ± 3.36 SEM%. Animals were euthanized by intraperitoneal injection of ketamine (100 mg/kg) and xylazine (20 mg/kg) at predetermined time points. Both eyes were collected for flat mount analysis from control animals sacrificed at p18 and from one eye of animals sacrificed at p32; the fellow eye was collected for immunohistochemistry, or retinas were snap frozen for later RNA or protein analysis. In total, 66 animals from 6 litters in 3 separate OIR experiments were included in this study. Only retinas with confirmed GFP expression were used, as described below in the retinal imaging section.

### Subretinal Injections

At the beginning of the 50% O_2_ cycle on p8, rat pups were given subretinal injections of lentiviral vector stock. General anesthesia was induced by inhalation of isoflurane, eye lids were carefully teased open and pupils were dilated using tropicamide eye drops 1% (Bausch + Lomb, Bridgewater, New Jersey). A 30 G cannula was used to pierce a pilot hole into the sclera near the limbus, a Hamilton syringe with a blunt 33 G cannula was advanced through the vitreous into the subretinal space and 1 µl lentivirus suspension (containing 10^6^ viral particles) in sterile PBS was slowly injected. Animals were randomly assigned to receive bilateral subretinal injections of vehicle (PBS), luciferase shRNA lentivirus (L-lucifshRNA), VEGF_164_ shRNA lentivirus (L-VEGF164shRNA) or VEGF-A shRNA lentivirus (L-VEGFAshRNA); both eyes received the same treatment. Pups were returned to their dams and replaced into the Oxycycler within 1.5 hours.

### Retinal Imaging

On p18 and p26, all animals were anesthetized using intraperitoneal ketamine and dexmedetomidine (75/0.5 mg/kg). Tropicamide eye drops 1% were applied to dilate pupils, and corneas were kept hydrated with 0.3% hypromellose gel (GenTeal Eye Gel, Novartis). Retinal images were recorded using a contact camera for small rodents (Micron IV, Phoenix Research Labs, Pleasanton, CA) under white light illumination to visualize the fundus. GFP-positive cells were imaged using a 469/35 nm exciter filter and a 488 nm long pass barrier filter. Animals were recovered by intraperitoneal injection of atipamezole (2.5 mg/kg).

### Electroretinograms

Rats were fully dark-adapted overnight and subsequently handled under dim red light illumination. Anesthesia was induced by intraperitoneal injection of ketamine and xylazine (75/10 mg/kg body weight). Pupils were dilated with tropicamide eye drops 1% and corneas lubricated with coupling gel (GenTeal Eye Gel, Novartis). Animals were positioned on a heated pad to maintain constant body temperature. Subdermal electrodes were used as the ground electrode inserted at the base of the tail and as the reference electrode placed between the eyes.

The focal ERG was recorded using the Image-Guided Focal ERG and Micron IV (Phoenix Research Labs). In order to allow focal ERGs to be measured in both eyes in some animals, recordings were either made on p28 or p29. Since animals were dark-adapted, previous retinal images of GFP were recorded to assure stimuli were only obtained in the area of GFP fluorescence. All focal ERG measurements were performed 48 hours after Micron imaging so as not to be influenced by prior bleaching of the photoreceptors during imaging. The objective containing the recording electrode was carefully advanced near the cornea and the retinal image was focused under red light illumination (850 nm). The recording area (aiming spot size A, 0.5 mm spot diameter) was chosen in a central part of the retina approximately one disc diameter away from the optic nerve head, which contained GFP-positive cells (Supplemental Fig. 2a). The focal ERG was recorded using LabScribeERG 3 software (Phoenix Research Labs) in response to white light flashes (5 ms duration, luminances ranging from −0.40 to 3.20 candela per square meter [cd s m^−2^], with 20 sweeps and 10 second intervals for −0.40 to 1.40 cd s m^−2^, 10 sweeps and 20 second intervals for 2.00 cd s m^−2^, 3 sweeps and 60 second intervals for 2.60 cd s m^−2^ and 2 sweeps and 2 second intervals for 3.20 cd s m^−2^). Focal ERGs were recorded in 7 eyes treated with L-lucifshRNA, 13 eyes with L-VEGF_164_shRNA and 10 eyes with L-VEGFAshRNA. Scattered light did not contribute to the focal ERG, as confirmed by a recording from the optic nerve head at the highest light intensity (Supplemental Fig. 2b).

An LKC UTAS Visual Diagnostic System with BigShot Ganzfeld was used to record the Ganzfeld ERG. Measurements were made either on p31 or p32 to allow recordings to be made in both eyes in some animals. A corneal contact lens electrode was positioned and the ERG was recorded using white light flashes (5 ms duration, luminances ranging from −6.1 to 2.20 cd s m^−2^, with 30 sweeps and 2 second intervals for −6.1 to −3.6 cd s m^−2^, 3 sweeps and 10 second intervals for −2.4 cd s m^−2^, 3 sweeps and 20 second intervals for −2.0 cd s m^−2^, 3 sweeps and 30 second intervals for −1.6 cd s m^−2^, 3 sweeps and 40 second intervals for −1.2 cd s m^−2^, 3 sweeps and 60 second intervals for −0.2 cd s m^−2^, 2 sweeps and 120 second intervals for 1.2 to 2.2 cd s m^−2^). Ganzfeld ERGs were recorded in 10 eyes treated with L-lucifshRNA, 12 eyes with L-VEGF_164_shRNA and 11 eyes with L-VEGFAshRNA.

Comparison to previously published data demonstrates that the a- and b-wave amplitudes of the focal and full-field ERGs were reduced in L-lucifshRNA treated retinas compared to untreated eyes and following subretinal injection of PBS. This finding provides evidence that ERG recordings were made in transduced areas of the retina^26^.

### Optical Coherence Tomography (OCT)

Anesthesia was administered by ketamine/dexmedetomidine (75/0.5 mg/kg) on p30, pupils were dilated with tropicamide eye drops 1% and coupling gel (GenTeal Eye Gel, Novartis) was applied to maintain corneal hydration. OCT was recorded in all GFP positive eyes using the OCT attachment for the Micron IV camera (Phoenix Research Labs). A region containing GFP-positive cells approximately one disc diameter away from the optic nerve head was chosen and a B-scan over 1620 μm with 1024 A-scans (at a distance of 1.5 μm) was recorded using OCT software (Phoenix Research Labs). GFP fluorescence was recorded simultaneously with OCT images. Anesthesia was reversed by intraperitoneal injection of atipamezol (2.5 mg/kg). Total retinal thickness and thickness of individual retinal layers was measured using InSight software (Phoenix Research Labs). Only high quality OCT images and areas of GFP fluorescence were included for OCT analysis, which resulted in measurements over 293–1620 μm (average 787 μm), which corresponds to 205–1024 A-scans. The thicknesses of the photoreceptor and outer nuclear layers (PRS + ONL), inner nuclear layers (INL) and the inner plexiform and ganglion cell layers (IPL + GCL) were determined and measurements along the B-scan were averaged for each animal. Thickness measurements were performed on 4 eyes treated with L-lucifshRNA, 6 eyes with L-VEGF_164_shRNA and 4 eyes with L-VEGF-AshRNA was included in the OCT analysis.

### Immunohistochemistry (IHC) and flat mounts

For immunohistochemistry, eyes were enucleated, fixed for 1 hour in 4% PFA in PBS, cryoprotected for 1 hour in 10% sucrose and overnight in 20% sucrose, embedded in a 2:1 mixture of 30% sucrose:optimal cutting temperature compound (Tissue-Tek) on dry ice. 12 μm cross sections were cut using a Leica CM3050S cryostat. Cryosections were then washed in PBS and incubated in a 0.1% Triton X-100 solution, to allow penetrance of DAPI stain, before mounting with DAPI Fluoromount-G (SouthernBiotech, Birmingham, AL) and imaged on an Olympus IX81 confocal microscope. From each treatment, a minimum of four sections were sampled from three retinas from three different rats. To insure comparable sampling between treatments, images were captured between 1 and 2 optical fields from the optic nerve head. Thickness of the photoreceptor segments and outer nuclear layer (PRS + ONL), inner nuclear layer (INL) and inner plexiform and ganglion cell layer (IPL + GCL) were determined using ImageJ. From each image four measurements were averaged, resulting in a total of at least 16 values for each layer from each of the three eyes per treatment condition.

For flat mounts, eyes were enucleated, fixed for 30 min in 4% PFA in PBS and overnight in HistoChoice tissue fixative (Sigma Aldrich), and washed 3 times in PBS. The retina was dissected free, blocked with 10% goat serum in 0.5% Triton X-100 in PBS, incubated with isolectin GS-IB4 conjugated with Alexa Fluor 488 for 48 hours and mounted with Fluoromount G mounting media (Southern Biotech, Birmingham, AL). Flat mounts were imaged using an Olympus IX81 microscope at a X4 magnification and stitched together using MetaMorph software ((Molecular Devices, Sunnyvale, CA). Areas of avascular area and intravitreal neovascularization were delineated using ImageJ software (NIH, Bethesda, MD), as displayed in Supplemental Fig. 3. All flat mounts were analyzed by two independent masked reviewers. In the occasional case of a discrepancy in defining a vascular feature, images were reviewed together to reach a consensus. Data are expressed as a percentage of avascular/total retinal area (AVA) or intravitreal neovascular/total retinal area (IVNV).

### Statistical Analysis

Using the rat OIR model to detect statistical significance with a power of 80% and a two-sided alpha of 0.05, a sample size of 9 flat mounts/group for AVA and 7/group for IVNV was needed. We therefore included at least 9 flat mounts for AVA and IVNV for the p32 time point. Pups from 3 different litters were used to address biologic diversity. A mixed effects linear regression for statistical comparisons of the experimental groups was used to account for clustering effects. OCT analysis of retinal thickness included four eyes treated with L-lucifshRNA, six with L-VEGF164shRNA and four with L-VEGFAshRNA. Retinal thickness by IHC was determined in at least four sections/eye from three eyes, taken from animals from three different litters for each experimental condition. Ganzfeld ERGs were recorded in 10 eyes for L-lucifshRNA, 12 eyes for L-VEGF164shRNA and 11 eyes for L-VEGFAshRNA. Focal ERGs were recorded in 7 eyes for L-lucifshRNA, 13 eyes for VEGF_164_ shRNA and 10 eyes for VEGF-A shRNA.

To avoid clustering effects by litter, pups nursed by the same dams were randomized to the treatment groups. Clustering remained for IHC, for which multiple images were used for analysis from each animal, and for Ganzfeld and focal ERG, where both eyes were used in some pups and was accounted for using the mixed effects linear regression model. In Ganzfeld and focal ERGs, for which recordings were made at several light intensities, we used a linear regression model with a multiple comparison adjustment for the three highest light intensities. For graphs, SEMs were determined using post-test marginal estimation from the mixed effects model to account for data clustering. For statistical modeling Stata-14 statistical software (College Station, TX, StataCorp LP) was used. Results were presented as mean ± SEM.

## Electronic supplementary material

Supplementary Information
