# Supplementary material for: Targeted Knockdown of Overexpressed VEGFA or VEGF164 in Müller cells maintains retinal function by triggering different signaling mechanisms
Source: Sci Rep. 2018 Jan 31;8:2003. doi: 10.1038/s41598-018-20278-4 (PMC5792486; doi:10.1038/s41598-018-20278-4)
Supplement: Supplementary file 1 — Supplementary Information [file 41598_2018_20278_MOESM1_ESM.pdf]

Targeted Knockdown of Overexpressed VEGFA or VEGF164 in Müller cells maintains retinal function by triggering different signaling mechanisms

Authors: Silke Becker[1†], Haibo Wang[1†], Aaron B. Simmons[1], Thipparat Suwanmanee[3], Gregory J. Stoddard[2], Tal Kafri[3], M. Elizabeth Hartnett[1*]

Supplemental Figure 1

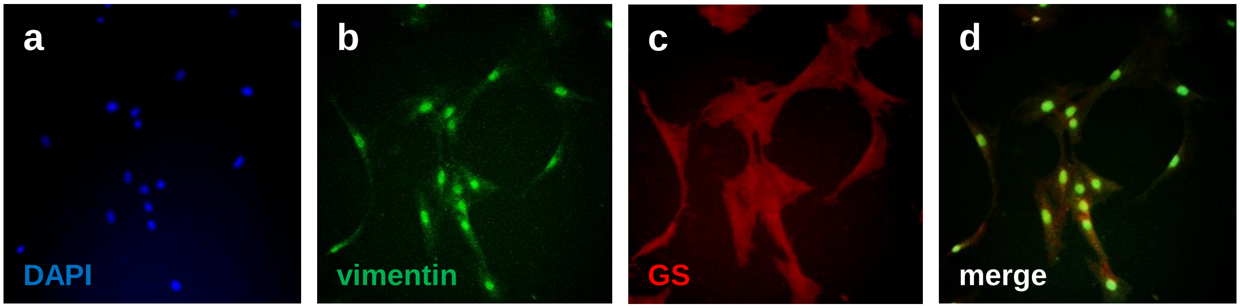

Supplemental Figure 1: Cultured rat Müller cells stained positive for the Muller glial cell markers glutamine synthetase and vimentin.

Supplemental Figure 2

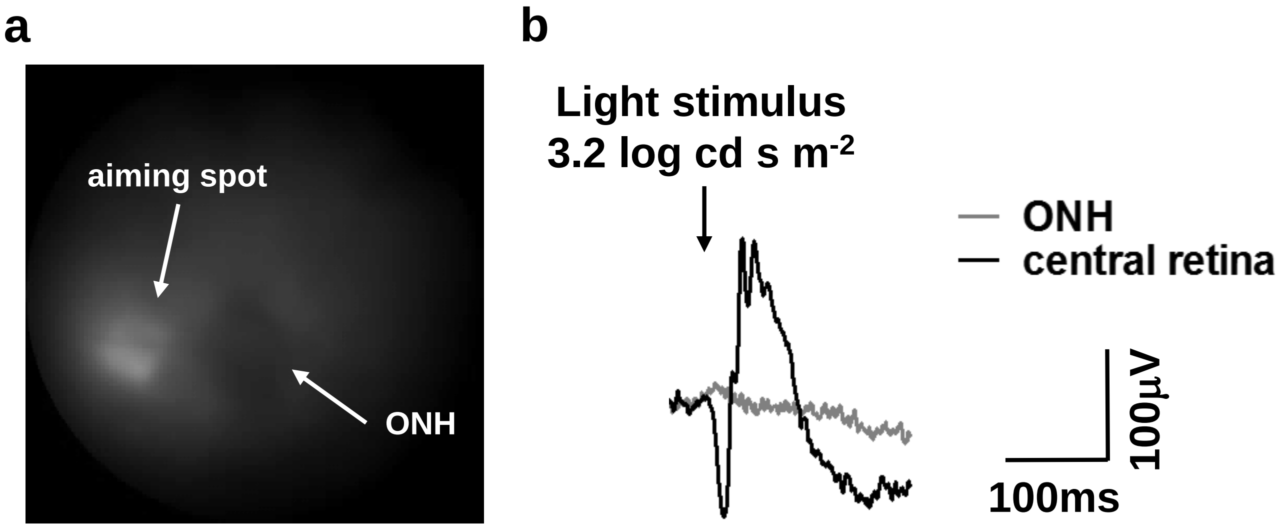

Supplemental Figure 2: a. The region (0.5mm spot diameter) on the neural retina approximately one disc diameter away from the optic nerve head was chosen to record the focal ERG. **b.** Absence of the ERG signal when light was focused on the optic nerve head (ONH) indicates that scattered light did not contribute to ERG recordings.

Supplemental Figure 3

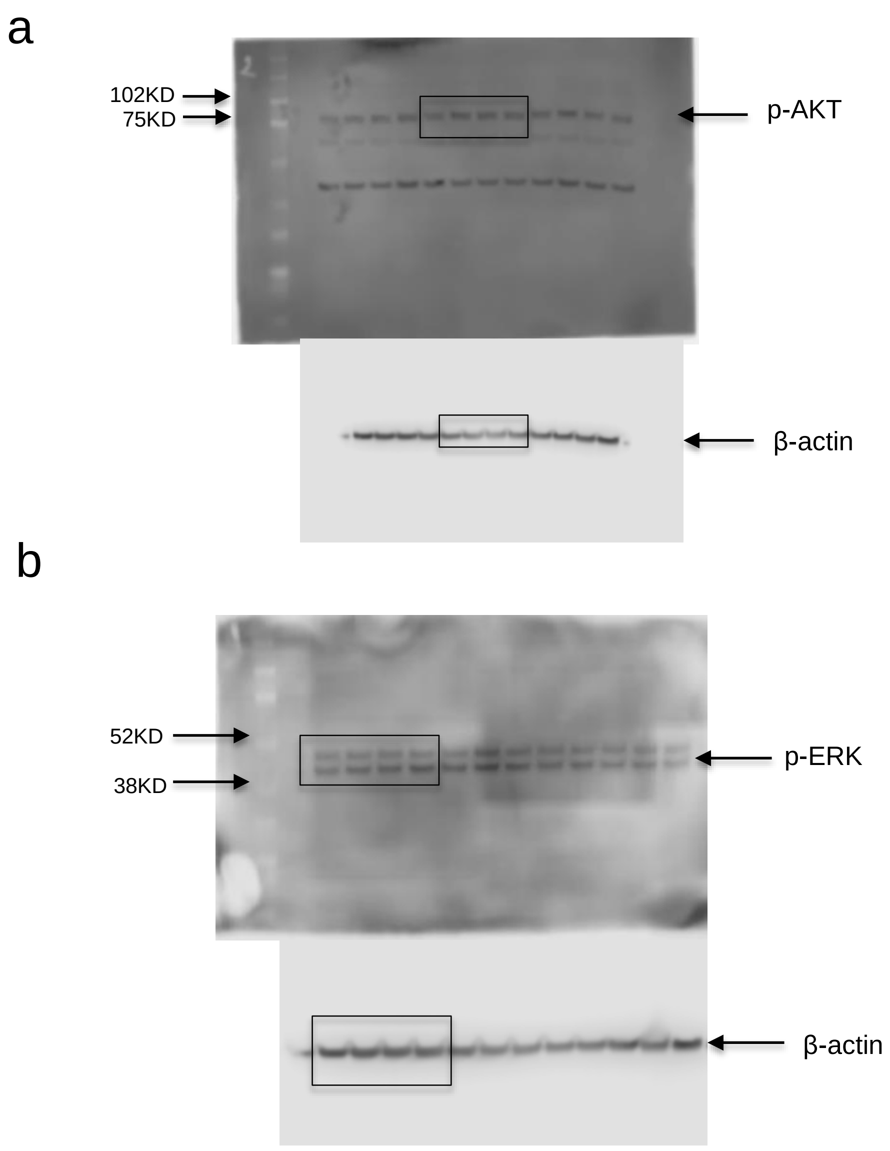

Supplemental Figure 3: a. Full length gels for p-AKT and β-actin; **b.** full length gels for p-ERK and β-actin (Boxes refer to the places from which the gels presented in Figure 8 were cropped).
